# Direct intracranial invasion of eccrine spiradenocarcinoma of the scalp: a case report and literature review

**DOI:** 10.1186/s12883-022-02749-4

**Published:** 2022-06-18

**Authors:** Yuji Kibe, Kuniaki Tanahashi, Kazuhiro Ohtakara, Yuka Okumura, Fumiharu Ohka, Kazuhito Takeuchi, Yuichi Nagata, Kazuya Motomura, Sho Akahori, Akihiro Mizuno, Hiroo Sasaki, Hiroyuki Shimizu, Junya Yamaguchi, Tomohide Nishikawa, Kenji Yokota, Ryuta Saito

**Affiliations:** 1 Department of Neurosurgery, Nagoya University Graduate School of Medicine, 65 Tsurumai-cho, Showa-ku, Nagoya, Aichi 466-8550 Japan; 2 Department of Radiation Oncology, Kainan Hospital, Aichi Prefectural Welfare Federation of Agricultural Cooperatives, Yatomi, Aichi Japan; 3 Department of Pathology and Laboratory Medicine, Nagoya University Graduate School of Medicine, Nagoya, Aichi Japan; 4 Department of Dermatology, Nagoya University Graduate School of Medicine, Nagoya, Aichi Japan

**Keywords:** Spiradenocarcinoma, Malignant spiradenoma, Scalp tumor, Intracranial invasion, Case report

## Abstract

**Background:**

Eccrine spiradenocarcinoma (SC), also known as malignant eccrine spiradenoma, is a rare malignant cutaneous adnexal neoplasm arising from long-standing benign eccrine spiradenoma. Malignant skin tumors rarely show direct intracranial invasion. However, once the intracranial structure is infiltrated, curative excision with sufficient margins can become extremely difficult, particularly when the venous sinuses are involved. No effective adjuvant therapies have yet been established. Here, we report an extremely rare case of scalp eccrine SC with direct intracranial invasion, which does not appear to have been reported previously.

**Case presentation:**

An 81-year-old woman presented with a large swelling on the parietal scalp 12 years after resection of spiradenoma from the same site. The tumor showed intracranial invasion with involvement of the superior sagittal sinus and repeated recurrences after four surgeries with preservation of the sinus. The histopathological diagnosis was eccrine SC. Adjuvant high-precision external beam radiotherapy (EBRT) proved effective after the third surgery, achieving remission of the residual tumor. The patient died 7 years after the first surgery for SC.

**Conclusions:**

Scalp SC with direct intracranial invasion is extremely rare. Radical resection with tumor-free margins is the mainstay of treatment, but the involvement of venous sinuses makes this unfeasible. High-precision EBRT in combination with maximal resection preserving the venous sinuses could be a treatment option for local tumor control.

## Background

Eccrine spiradenocarcinoma (SC), also known as malignant eccrine spiradenoma, is a rare malignant cutaneous adnexal neoplasm arising from long-standing benign spiradenoma [1]. A history of trauma to preexisting spiradenoma has been reported [1]. A connection to Brooke-Spiegler Syndrome, an autosomal-dominant genetic disorder phenotypically characterized by multiple skin tumors such as spiradenomas, cylindromas, trichoepitheliomas and tumors of the parotid gland, is also suspected [1, 2]. In 1971, Dabska provided the first description of a case of malignant transformation of eccrine spiradenoma [3]. The most common locations are the extremities, although reports have also described occurrences on the face, scalp and chest. According to a review of the literature, median patient age is 60 years (range, 8–92 years) and the sex distribution is balanced [2]. The most common symptoms are accelerated growth, pain, and ulceration, typically present for more than 2 years. Metastatic spread to lung, bone, lymph nodes, liver, kidney, and breast has been documented [2, 4]. SC with distant metastasis progresses aggressively and the prognosis is dismal [4]. SC commonly shows the key histopathological features of spiradenoma, comprising dermal basaloid islands with multiple cuticle-lined ducts sprinkled with lymphocytes, although specific diagnostic markers have yet to be identified. Morphological grading is reportedly associated with the clinical course. The only recommended treatment is wide local excision with tumor-free margins. The efficacies of adjuvant chemotherapy or radiotherapy have yet to be established.

Malignant skin tumors rarely show direct intracranial invasion. However, once the intracranial structure is infiltrated, wide local excision with sufficient margins can become unavailable, particularly when the venous sinuses are involved. Radical treatment can thus become difficult without effective adjuvant therapy. Here, we report an extremely rare case of scalp eccrine SC that showed direct intracranial invasion, repeated recurrences after surgery, and the effectiveness of adjuvant high-precision external beam radiotherapy (EBRT), which does not appear to have been reported previously.

## Case presentation

### Patient information

**Fig. 1:**
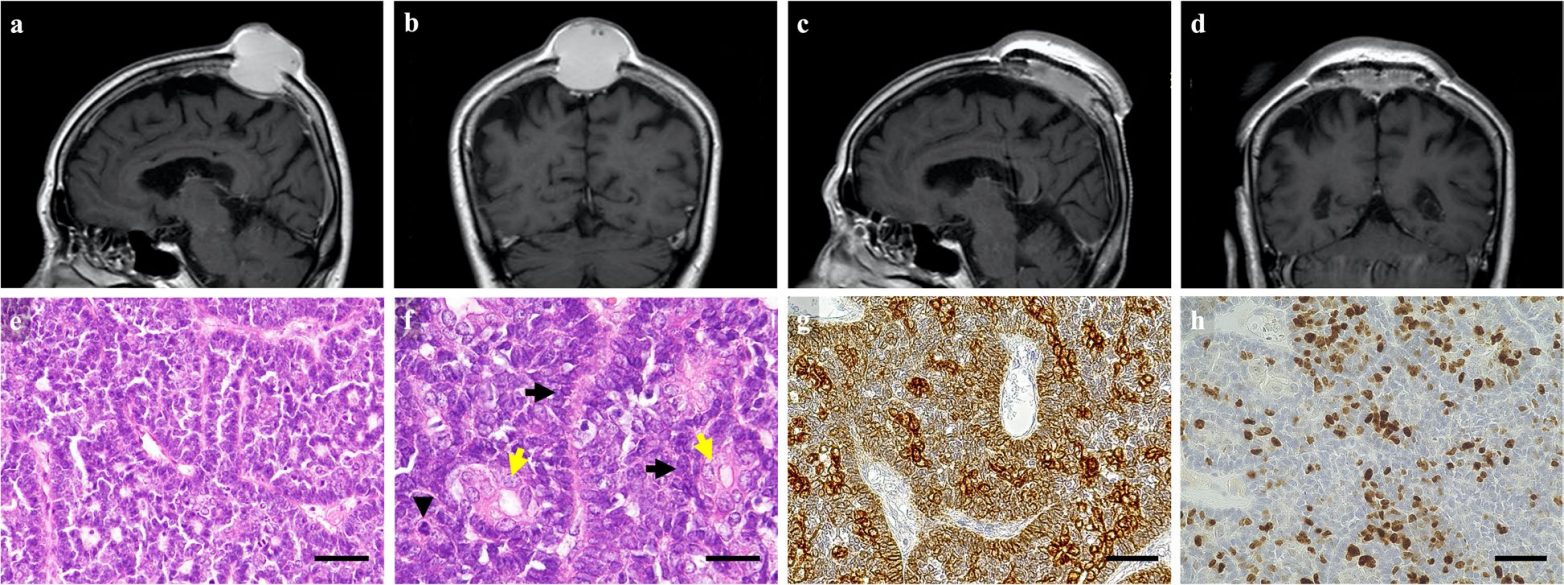
Magnetic resonance imaging before and after first surgery and histopathological images. **a–d** A single large, cystic mass is invading the parietal bone bilaterally and shows attachment to the superior sagittal sinus without occlusion. The tumor measures 41 × 42 × 34 mm (**a** sagittal view; **b** coronal view). The cystic lesion is resected, preserving the superior sagittal sinus and dura. The cranium and scalp are reconstructed with titanium mesh and local pedicled skin flap (**c** sagittal view; **d** coronal view).** e–h** Histopathological images obtained from first surgery. Hematoxylin and eosin staining demonstrates that glandular lumens and ducts are forming palisading or solid nests. Bar = 50 μm (**e**). Two distinct cell types, with eosinophilic cuticular cells (yellow arrows) surrounded by poroid cells with a high nuclear-cytoplasmic ratio (black arrows) are shown. Overall cytoplasmic atypia, some mitotic figures (black arrowhead) and partial loss of the two-cell structure are also observed, compatible with low-grade spiradenocarcinoma. Bar = 25 μm (**f**). Immunohistochemistry shows broad expression of cytokeratin 7. Bar = 50 μm (**g**). Ki-67 labeling index is approximately 30%. Bar = 50 μm (**h**). The histopathological images are obtained using the Olympus BX51 microscope and Olympus DP21 digital microscopy camera (Olympus Corporation, Tokyo, Japan).

An 81-year-old woman, who had a history of resection of a benign eccrine spiradenoma from the parietal scalp 12 years earlier, presented with a large swelling on the same region that had been growing over the preceding 5 years without any neurological deficit. Radiological examinations demonstrated that the lesion was a single, large cystic mass invading bilateral parietal bones and attached to but not occluding the superior sagittal sinus (SSS) (Fig. 1a, b). Whole-body positron emission tomography revealed no lymph node or distant metastases.

### First operation, diagnosis and postoperative course

The lesion was excised along with surrounding scalp and bone, and detached from the SSS. The dura mater covering the SSS was electrocoagulated. Reconstruction of the removed skull and scalp was then performed using titanium mesh and a pedicled skin flap from the occipital region which was replaced by a skin graft from the femoral area (Fig. 1c, d). Histopathological examination revealed the features of low-grade eccrine SC. Two distinct cell types were observed, comprising eosinophilic cuticular cells surrounded by poroid cells with a high nuclear-cytoplasmic ratio forming palisading or solid nests, accompanied by glandular lumens and ducts. Overall cytoplasmic atypia, some mitotic figures and partial loss of the two-cell structures were also observed (Fig. 1e, f). Cytokeratin 7 was broadly expressed, and Ki-67 labeling index was approximately 30% (Fig. 1g, h). Close follow-up was continued considering the risk of recurrence.

### Second operation, diagnosis and postoperative course

**Fig. 2:**
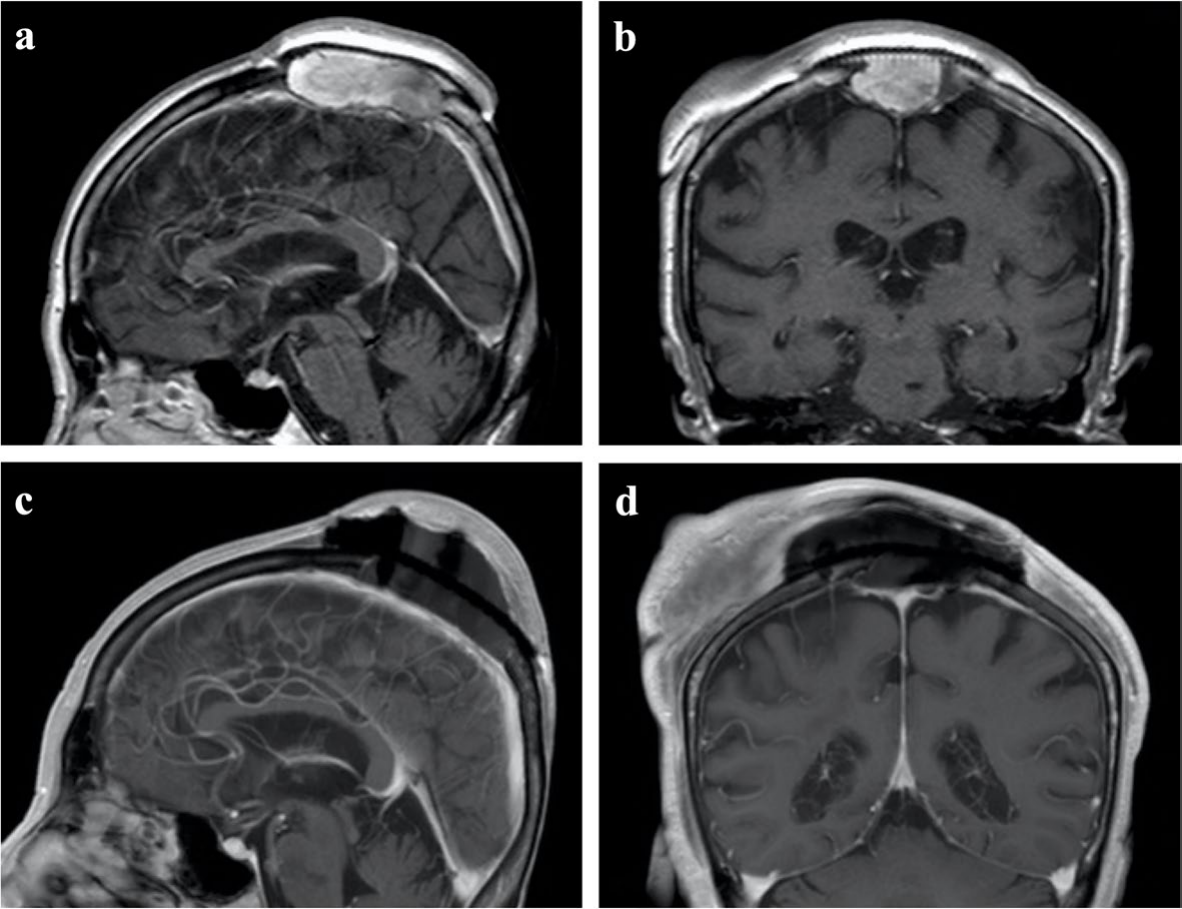
Magnetic resonance imaging before and after second surgery**.** Recurrence of solid tumor is seen at the superior sagittal sinus (**a** sagittal view; **b** coronal view). The tumor is totally excised, preserving the SSS, adjacent dura and pedicled scalp flap (**c** sagittal view; **d** coronal view)

Thirty-three months after the first surgery, solid tumor was seen to have recurred in the SSS and a second resection was performed (Fig. 2a, b). The tumor was totally excised, again preserving the SSS, adjacent dura and pedicled scalp flap (Fig. 2c, d). Histopathological diagnosis was similar to the previous lesion, other than the loss of the large cyst formation. No adjuvant therapy was administered, in consideration of the lack of evidence of efficacy, the age of the patient, and a recent history of ischemic brainstem infarction prior to the second surgery.

### Third operation, diagnosis and postoperative treatment

**Fig. 3:**
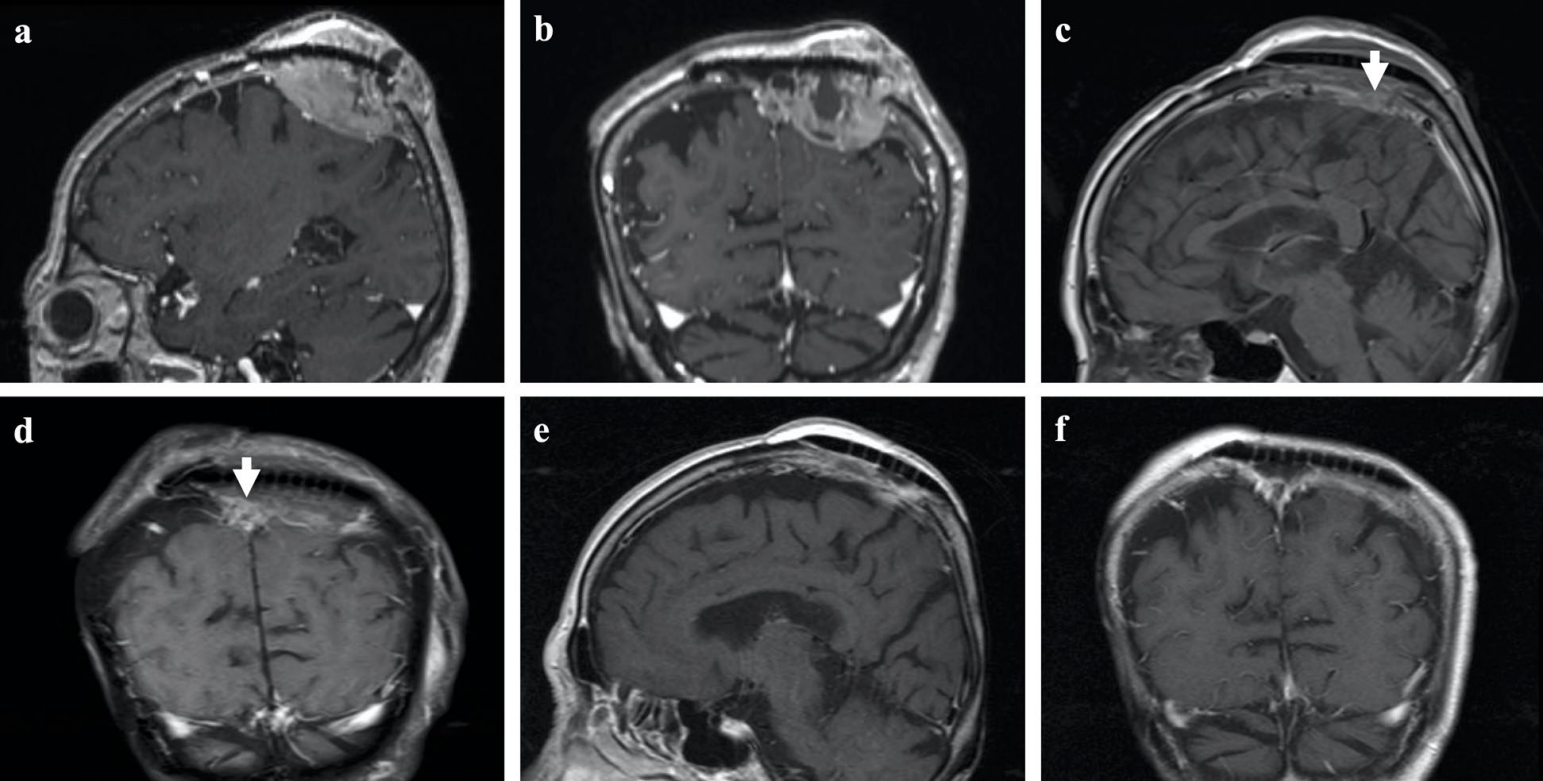
Magnetic resonance imaging before and after third surgery**.** A heterogeneous, gadolinium-enhancing tumor is observed in the epidural region with extracranial extension pushing against the titanium mesh. The SSS is nearly compressed but is not occluded, and the scalp is about to rupture (**a** sagittal view; **b** coronal view). The recurrent tumor is resected with the skin flap, titanium mesh and surrounding bone edge. Tumor invading the SSS is not resected (arrows). An artificial bone flap made from polyethylene is used for cranioplasty. Scalp reconstruction is made with a new pedicled skin flap (**c** sagittal view; **d** coronal view). The residual tumor shows rapid shrinkage and has vanished by 3 months after radiotherapy (**e** sagittal view; **f** coronal view)

Twelve months after the second surgery, a heterogeneous contrast-enhancing mass was observed in the epidural region with extracranial extension pushing against the titanium mesh. The SSS was compressed but not occluded, and the scalp skin was about to rupture (Fig. 3a, b). No metastatic lesion was evident on whole-body examination.

The third operation was performed with resection of the skin flap, titanium mesh, and surrounding bone edge. The tumor invading the SSS could not be resected due to marked bleeding. An artificial bone flap made from polyethylene was used for the cranioplasty instead of metallic material, which would reduce the scattered radiation dose to the surrounding tissue. Scalp reconstruction was performed with a new pedicled skin flap from the left temporal region (Fig. 3c, d). The histopathological diagnosis was compatible with the first lesion. Two months after the third surgery, the patient received 6 MV X-ray EBRT using simultaneous integrated boost (SIB) volumetric-modulated arc therapy (VMAT) with the prescribed dose of 50 Gy in 25 fractions to the planning target volume margin (residual gross tumor invading the SSS + 5 mm, tumor cavity + 3 mm) as well as SIB with 57.5 Gy to the residual gross tumor margin. The median dose to the gross tumor volume was 60.4 Gy. The residual tumor rapidly shrank and had vanished by 3 months after completion of the EBRT (Fig. 3e, f).

### Fourth operation, diagnosis and postoperative treatment

**Fig. 4:**
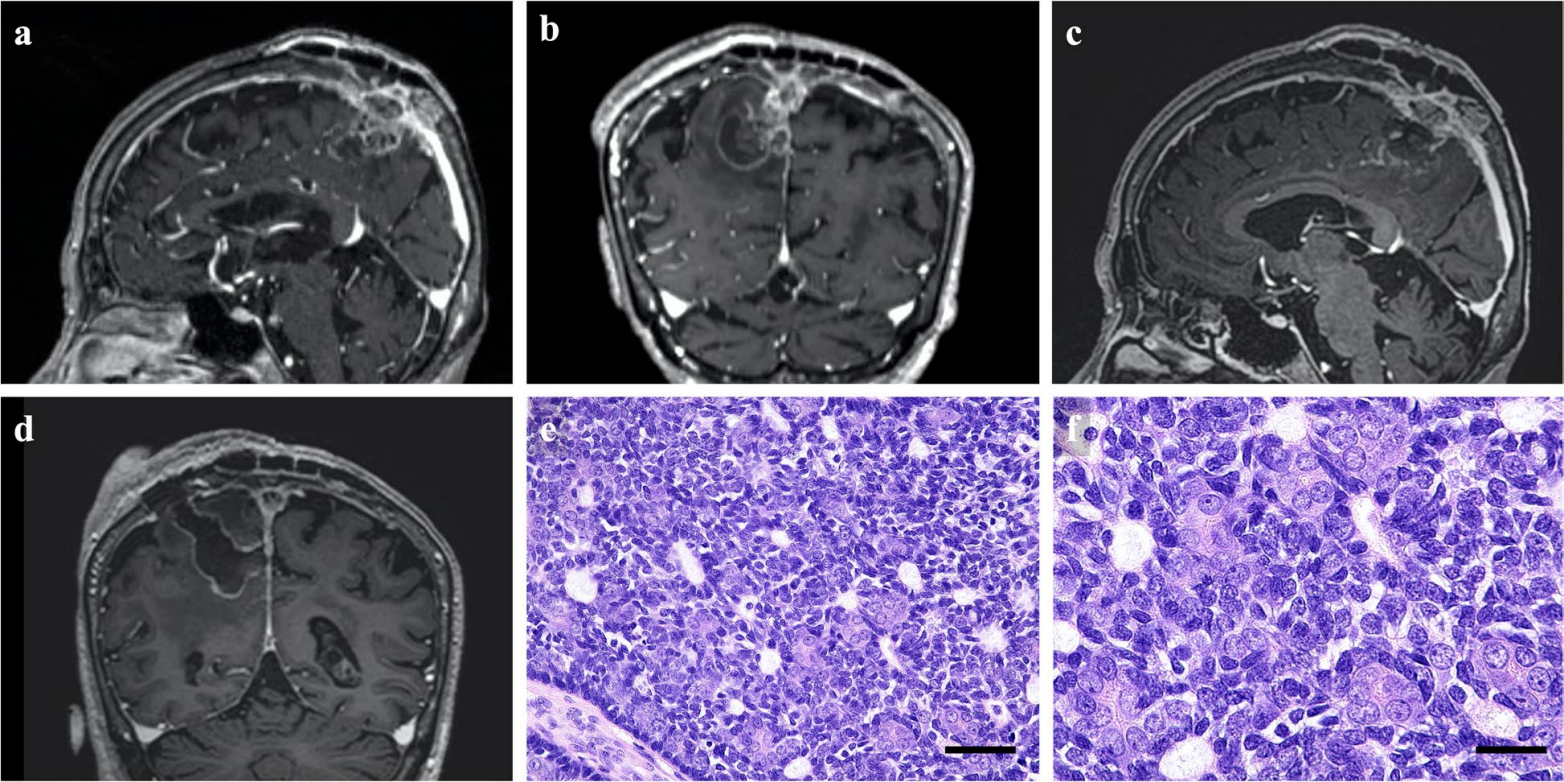
Magnetic resonance imaging before and after fourth surgery and histopathological images.** a–d** Tumor recurrence is observed with intracerebral extension arising from the SSS forming a cystic lesion (**a** sagittal view; **b** coronal view). Palliative endoscopic partial resection of the cystic lesion is performed (**c** sagittal view; **d** coronal view). **e**, **f** Histopathological images obtained from fourth surgery. Hematoxylin and eosin staining shows the features of low-grade spiradenocarcinoma observed in Fig. 1 have been retained. Bar = 50  μm (**e**). Bar = 25  μm (**f**). The histopathological images are obtained using the same equipment as used in Fig. 1

Twenty months after the third surgery, the patient developed left hemiparesis and a recurrent tumor was observed with intracerebral extension arising from the SSS forming a cystic lesion (Fig. 4a, b). The patient concurrently developed rectal carcinoma with lower gastrointestinal bleeding. Palliative endoscopic partial resection of the cystic lesion was performed, followed by stereotactic radiotherapy using CyberKnife^®^ (Accuray Incorporated, Sunnyvale, CA) with 42.2 Gy in 10 fractions to the margin of the residual cyst wall and the tumor involving the SSS (Fig. 4c, d). Histopathological examination demonstrated that the features of low-grade SC had been sustained (Fig. 4e, f).

The patient was transferred to another hospital and died 7 months after the last treatment, 7 years after the first surgery, with no apparent swelling of the scalp.

## Discussion and conclusions

Eccrine SC is a rare malignant cutaneous adnexal neoplasm arising from benign spiradenoma of the eccrine sweat gland [1]. Staiger reported that in SC cases, recurrence-free survival, tumor recurrence and death were seen in 37.2% (45/121), 17.4% (21/121) and 10.2% (13/121), respectively, with a median follow-up of 24 months [2]. Metastatic spread to multiple organs has been documented. The overall prognosis of eccrine SC is poor. In a meta-analysis of eccrine SCs, 24 patients harboring distant metastasis showed a median survival of 16 months with limited efficacy from adjuvant chemotherapy and/or EBRT, while 35 cases without distant metastasis treated with local resection achieved a disease-free survival rate of 100% within a mean follow-up of 33 months [4]. An aggressive surgical approach for SC is therefore supported in the absence of metastasis.

Five cases of eccrine SC of the scalp have been reported previously (Table 1) [5–9]. All cases were primarily treated with excision and skin grafting. Three showed no recurrence or distant metastasis [7–9]. One developed metastasis to the neck, causing compression of the sixth cervical vertebral body [6]. Another showed local recurrence and metastases to the lung, liver and pelvis, which were treated with chemotherapy in the form of epirubicin, cisplatin and capecitabine, but showing no regression [5]. Those two cases had involved relatively large primary lesions.

**Table 1:** Eccrine spiradenocarcinoma of the scalp

Author	Year	Age (years)	Sex	Location	History	Size (cm)	Primary treatment	Secondary treatment	Time to Rec	Follow-up	Site of Rec	Status
Jamshidi [6]	1999	72	female	scalp	many years	12	Res	-	2 years	2 years	Distant	Alive
Beekley [8]	1999	60	female	parietal	> 2 years	1.5 × 1.9 × 1.2	Res 3 times	-	-	14 months	-	Alive
Russ [9]	2002	65	male	parietal	30–40 years	2	Res	-	-	12 months	-	Alive
Seyhan [7]	2008	27	female	parietal	20 years	6 × 6 × 1.5	Res	-	-	24 months	-	Alive
Chow [5]	2014	37	male	parietal	a few months	6 × 4	Res	chemotherapy	10 months	NA	Local, distant	Alive

Eccrine adenocarcinoma or malignant eccrine tumor, not specified as eccrine spiradenocarcinoma or malignant eccrine spiradenoma, is excluded

*NA*, not available; *Rec*, recurrence; *Res*, resection; *-*, not applicable

The present report describes the first case of scalp SC with intracranial extension, as the five cases described above showed no intracranial extension. To the best of our knowledge, only one case report has described eccrine SC with direct intracranial extension [10]. That case involved a large exophytic tumor on the face invading the middle cranial fossa. Craniotomy and debulking of the tumor were performed, then the patient was transferred to a palliative care team and died 5 weeks after diagnosis.

Our search of the literature identified 20 cases of scalp sweat gland tumors with intracranial invasion (Table 2) [10–28]. These consisted of four benign tumors and 16 malignant tumors. Hidradenocarcinoma was the most common pathology, with 5 cases, and only one case was SC. Tumors were attached to the SSS in 7 cases, sigmoid sinus in 2 cases, confluence of sinuses in 1 case and transverse sinus in 1 case. The venous sinuses were preserved in all cases. In most cases, primary treatment was surgical resection. Eleven cases received radiotherapy (prescribed dose range, 20–60 Gy) including four cases that were administered chemotherapy, and one case that received an unspecified “adjuvant therapy”. None of tumor size, depth of extension, venous sinus involvement or adjuvant therapy were associated with recurrence in this limited number of cases. Nine cases developed local and/or distant recurrence. Even two non-malignant cases showed local recurrence, as did the present case, implying the necessity of careful follow-up.

**Table 2:** Sweat gland tumors with intracranial invasion

Author	Year	Age (years)	Sex	Location	Size (cm)	Skin destruction	Depth of extension	Sinus involvement	History	Histopathology	Primary treatment	Salvage treatment	Time to Rec	Follow-up	Site of Rec	Status
Bradbury [15]	1984	79	Male	External auditory meatus	3 × 4	-	Intracerebral	-	13 years	Hidradenoma	Res (intradural)	-	-	1 year	Local	Alive
Urbanski [24]	1985	68	Female	Parietal	grapefruit-size	-	Epidural; intracerebral	-; TS	many years	Cylindroma	Res with scalp, skull	2nd Res with skull and dura; 3rd Res	< 1 year; < 24 months	3 years	Local	Alive
Sridhar [26]	1989	60	Female	NA	NA	NA	Skull; epidural and intracerebral (multiple metastases)	NA	NA	Eccrine adenocarcinoma	Chemo	Chemo (3 regimens); WBRT (20 Gy), Chemo	35 months; 5 months; NA	84 months	Distant	Dead
Veillon [18]	1996	61	Female	Ethmoid sinus	8 × 5	-	Epidural	-	6 months	Adenoid cystic carcinoma (cylindroma)	Res	-	-	6 months	-	Alive
Wyld [27]	1996	76	Female	Parietal	8	-	Epidural	SSS	3 months (familial cylindromatosis)	Dermal cylindroma	Res with scalp and skull	-	-	23 years	-	Alive
Sigal [13]	1997	54	Female	Retroauricular	6 × 4.5	+	Subdural	SS	10 years	Eccrine porocarcinoma	Res, Neck dissection RT (50 Gy to cervical region, 45 Gy to tumor site)	Chemo	NA; subsequent months	2.5 years	Local	Dead
Ritter [17]	1999	82	Male	Occipital	5 × 5.3	-	Epidural	SSS	4 years	Eccrine porocarcinoma	Res, RT (60 Gy)	-	-	1 year	-	Alive
Castro [28]	2000	39	Female	Parietal	NA	-	Epidural	-	2 years	Ceruminous adenoid cystic carcinoma	Res, RT (60 Gy)	-	-	1 year	-	Alive
Ohta [25]	2004	73	Female	Frontal	6 × 4.5 × 5.5	+	Subdural	SSS	> 20 years	Microcystic adnexal carcinoma	palliative Res	-	NA	NA	Local, distant	Dead
Donovan [14]	2006	54	Female	Parietal	11 × 13 × 5	+	Epidural	SSS	43 years	Eccrine adenocarcinoma	Res with skull	-	NA	18 months	NA	Dead
Durairaj [20]	2006	70	Female	Orbital	NA	-	NA	NA	NA	Malignant hidradenoma	Res, Chemo, RT	-	< 1 year	< 1 year	Distant	Dead
Gildea [12]	2007	59	Male	Parietal	9 × 7.9	-	Intracerebral	SSS	30 years (familial cylindromatosis)	Cylindroma	Res	-	NA	NA	NA	NA
Pedamallu [10]	2009	48	Female	Buccal	10 × 5	-	Intracerebral	-	-	Malignant eccrine spiradenoma	Res (partial)	-	NA	5 weeks	NA	Dead
Sheth [16]	2010	45	Female	Parietal	4.2 × 3.8	-	Intracerebral (multiple metastases)	-	6 years	Eccrine mucinous carcinoma	Res, Chemo	Res, WBRT	2.5 years	8 years	Distant	Dead
Lee [22]	2010	50	Female	Fronto-parietal	3.3 × 5.4 × 4.8	-	Intracerebral	SSS	1 year	Low-grade hidradenocarcinoma	Res, WBRT (54 Gy)	-	-	3 years	-	Alive
Araujo [19]	2012	31	Female	Occipital	NA	+	Intracerebral	Conflu	4 months	Malignant chonrdoid syringoma	Res with dura, adjuvant therapy	-	-	NA	-	NA
Maiti [21]	2014	14	Female	Parietal	NA	-	Epidural; intracerebral	-	4 years	Malignant nodular hidradenoma	Res	2nd Res with skull; 3rd Res, RT (45 G)	6 months; 1 year; 1 year	2 years 6 months	Local	Alive
Maiti [21]	2014	45	Female	Parietal	NA	-	Intracerebral	-	6 months	Malignant nodular hidradenoma	Res with skull, RT	-	< 3 months	< 3 months	NA	Dead
Jagannatha [23]	2016	76	Male	Parietal	7 × 5 × 4; 6 × 5 × 3	+	Intracerebral	SSS	14 years	Clear cell hidradenocarcinoma	Res with scalp, skull and dura, WBRT	-	-	24 months	-	Alive
Shen [11]	2019	37	Male	Occipital	3.3 × 1.8	-	Skull; subdural	-; SS	-	Eccrine porocarcinoma	Res with skull	Res with skull and dura, RT	7 months; 1 year	1 year 7 months	Distant	Alive

*Chemo,* Chemotherapy; *Conflu,* Confluence of sinuses; *NA,* Not Available; *Rec,* Recurrence; *Res,* Resection; *RT,* Radiotherapy; *SS,* Sigmoid Sinus; *SSS,* Superior Sagittal Sinus; *TS,* Transverse Sinus; *WBRT,* Whole-brain Radiotherapy; -, not applicable

In malignant skin tumors, wide local excision with 1-cm, tumor-free, circumferential and deep margins is recommended for primary treatment [4]. However, resection with the margins could be difficult in cases where the tumor shows intracranial invasion. In the present case, repeated tumor recurrence was seen after multiple surgical resections. Tumor-free margins were secured only in the scalp and skull. Tumor detachment from the SSS followed by electrocoagulation proved insufficient for tumor control. However, sacrifice of the venous sinuses, particularly the SSS, is expected to cause brain swelling, infarction, hemorrhage and even life-threatening conditions [29, 30]. If the venous sinus had been spared tumor involvement, dural resection with the tumor could have been performed to obtain a tumor-free margin.

Although radiotherapy is generally not recommended as the primary therapy for sweat gland carcinomas, due to their radioresistance [31], five cases of SC treated by radiotherapy have been reported [32–36]. Rebegea reported a case of SC in the femoral region with lymphatic metastasis treated using wide local excision and lymph node dissection, followed by 50 Gy of radiotherapy to the tumor cavity and inguinal lymphatic nodes, and six courses of chemotherapy comprising carboplatin and paclitaxel, resulting in postoperative recurrence-free survival of 3 years [32]. Tay reported a case of eccrine SC in the lower leg with lymphatic metastasis treated by wide local excision and lymph node dissection, followed by radiotherapy of 59.4 Gy to the tumor bed and 45 Gy to the inguinal and pelvic lymph nodes. Local recurrence occurred 9 months after completion of this treatment [33]. In the other three cases, details of radiation dose and outcomes were not reported [34–36].

In the case reported here, image-guided high-precision EBRT with the SIB-VMAT technique to deliver higher dose to the gross tumor was administered after the third surgery and tumor remission was attained within 3 months. No other reports have described the efficacy of photon EBRT. The SIB-VMAT prolonged the time to recurrence from 12 months (after the second surgery) to 20 months (after the third surgery). Notably, recurrence after the EBRT was limited to around the SSS, where unresectable tumor had been left, even though the tumor was seen to show broad attachment to the dura at the third operation. Immediate adjuvant EBRT after maximal resections like the first or second surgeries might have yielded longer, better tumor control [37], which was not performed in the present case. The fourth operation and second EBRT were palliative treatments and follow-up examinations were not conducted.

We have reported a rare case of scalp SC with direct intracranial invasion. Radical resection with tumor-free margins represents the mainstay of treatment, but involvement of the venous sinuses makes this method unfeasible. High-precision EBRT with sufficient tumor dose in combination with maximal resection preserving the venous sinuses could be a treatment option for longer tumor control.

## Data Availability

All data generated or analysed during this study are included in this published article.

## Abbreviations

EBRT: External beam radiotherapy
SC: Spiradenocarcinoma
SIB: Simultaneous integrated boost
SSS: Superior sagittal sinus
VMAT: Volumetric-modulated arc therapy
